# Accidental Endotracheal Tube Compromise During Genioplasty: An Anesthetic and Surgical Challenge

**DOI:** 10.7759/cureus.113240

**Published:** 2026-07-23

**Authors:** Prahlad Shetty, Shreyas Sorake, Rudragouda R

**Affiliations:** 1 Department of Oral and Maxillofacial Surgery, AJ Institute of Dental Sciences, Mangalore, IND

**Keywords:** endotracheal tube leakage, endotracheal tube exchange, genioplasty, perioperative complications, submental intubation, transection

## Abstract

Airway security is critical in maxillofacial surgery, where operative procedures are performed in close proximity to the airway. Although genioplasty is considered a safe adjunct to orthognathic surgery, inadvertent endotracheal tube (ETT) damage during osteotomy is a rare but potentially life-threatening complication.

A 22-year-old male with skeletal Class III malocclusion underwent combined orthognathic surgery with genioplasty under general anesthesia. During the genioplasty osteotomy, the ETT was partially transected, resulting in an acute airway emergency. The complication was promptly recognized through changes in ventilation parameters, and coordinated intervention by the anesthetic and surgical teams enabled successful airway control and tube replacement without adverse sequelae.

Intraoperative ETT injury during genioplasty is an uncommon but serious event. Early recognition, effective communication, and a well-coordinated multidisciplinary response are essential to prevent catastrophic airway compromise and ensure patient safety.

## Introduction

Airway management is fundamental to the practice of anesthesia, and its integrity must be maintained throughout the perioperative period. In maxillofacial procedures, the close proximity of the surgical site to the airway places the endotracheal tube (ETT) at risk of accidental damage. Although rare, ETT compromise caused by surgical instruments has been reported during procedures involving the mandible, maxilla, and chin region and can lead to sudden airway emergencies if not promptly recognized [1,2].

Genioplasty is frequently performed as an adjunct to orthognathic surgery for both aesthetic and functional correction. The osteotomy is carried out in close relation to the oral cavity and lower anterior facial structures, where the ETT, particularly during oral intubation converted to submental intubation, may lie vulnerable.

Airway management for orthognathic surgery may be achieved by nasotracheal intubation, by oral intubation with submental conversion, or, in prolonged or complex reconstructions, by tracheostomy. Nasotracheal intubation is conventional but becomes unsuitable when the tube would obstruct the operative field or interfere with the maxilla during a Le Fort osteotomy. Submental intubation, first described by Hernández Altemir in 1986 as an alternative to tracheostomy in maxillofacial trauma, exteriorizes an oral ETT through the floor of the mouth and submental triangle, affording an unobstructed field and free intraoperative access to the dental occlusion [3,4]. Although developed for panfacial trauma, it has since been applied to selected orthognathic procedures in which repeated occlusal assessment is required [5]. Instrument-related ETT injury during such shared-airway surgery is rare but well documented, with partial and complete transection reported during orthognathic and maxillary osteotomies [6-8].

The use of powered saws, osteotomes, and rotary instruments further increases the risk of inadvertent ETT injury during this procedure [9].

Accidental damage to the ETT during genioplasty is particularly hazardous because it may result in air leak, inadequate ventilation, aspiration of blood, and rapid deterioration in oxygenation. Partial tube damage may initially preserve ventilation, thereby delaying diagnosis until significant airway compromise develops.

We report a case of accidental ETT compromise during genioplasty in a patient undergoing combined Le Fort I advancement, bilateral sagittal split osteotomy (BSSO) setback, and genioplasty. To our knowledge, few reports specifically address instrument-related injury to a submentally routed tube during genioplasty; we highlight the anesthetic implications, intraoperative management, and preventive strategies relevant to this shared-airway setting.

## Case presentation

Patient profile

A 22-year-old male, weighing 68 kg, was diagnosed with skeletal Class III malocclusion and planned for elective orthognathic surgery consisting of Le Fort I osteotomy with maxillary advancement, bilateral sagittal split osteotomy for mandibular setback, and advancement genioplasty. The patient had no significant medical comorbidities and was classified as American Society of Anesthesiologists physical status I. Preanesthetic evaluation revealed normal airway parameters with Mallampati class I, adequate mouth opening, and normal neck mobility. Routine investigations were within normal limits. Written informed consent for surgery and publication of this case report was obtained from the patient. Notably, this reassuring airway assessment did not preclude the subsequent complication, which arose from within the surgical field rather than from any anatomical airway difficulty.

Anesthetic management

**Figure 1:**
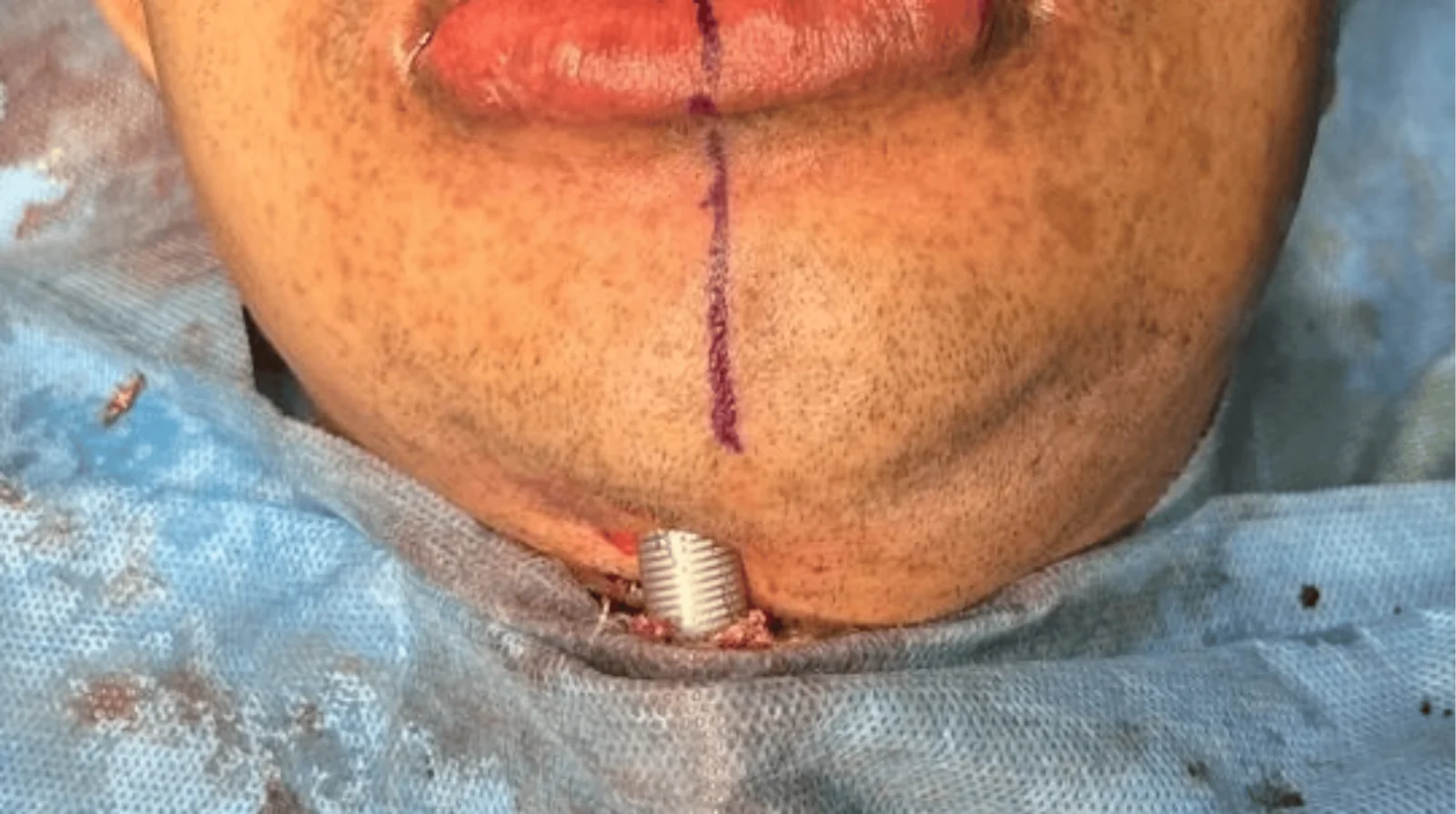
Clinical photograph demonstrating submental intubation with the endotracheal tube

The patient was premedicated and induced under standard monitoring, including electrocardiography, non-invasive blood pressure, pulse oximetry, and capnography. Following intravenous induction and neuromuscular blockade, oral intubation was performed with a cuffed 7.0 mm internal-diameter flexometallic (reinforced) ETT, which was subsequently converted to submental intubation to facilitate surgical access (Figure 1). Submental conversion was carried out using the standard Hernández Altemir technique: a short paramedian submental incision was made, blunt dissection was carried through the floor of the mouth into the oral cavity, and the temporarily disconnected tube, together with its pilot balloon, was delivered through the submental tunnel and reconnected [3,5]. Submental rather than nasotracheal intubation was selected on surgical grounds rather than for any airway difficulty: the planned Le Fort I osteotomy and the need for repeated, unobstructed intraoperative assessment of the dental occlusion make a nasotracheal tube liable to obscure the field and to be displaced or damaged during maxillary manipulation, whereas the patient's Mallampati class I airway posed no barrier to conventional intubation.

Correct placement was confirmed by bilateral chest auscultation and continuous capnography. The tube was secured with the submental segment retracted clear of, and positioned away from, the mandibular symphysis, and the oral cavity was packed. Anesthesia was maintained with inhalational agents in an oxygen-air mixture and intermittent doses of muscle relaxant.

Intraoperative event

**Figure 2:**
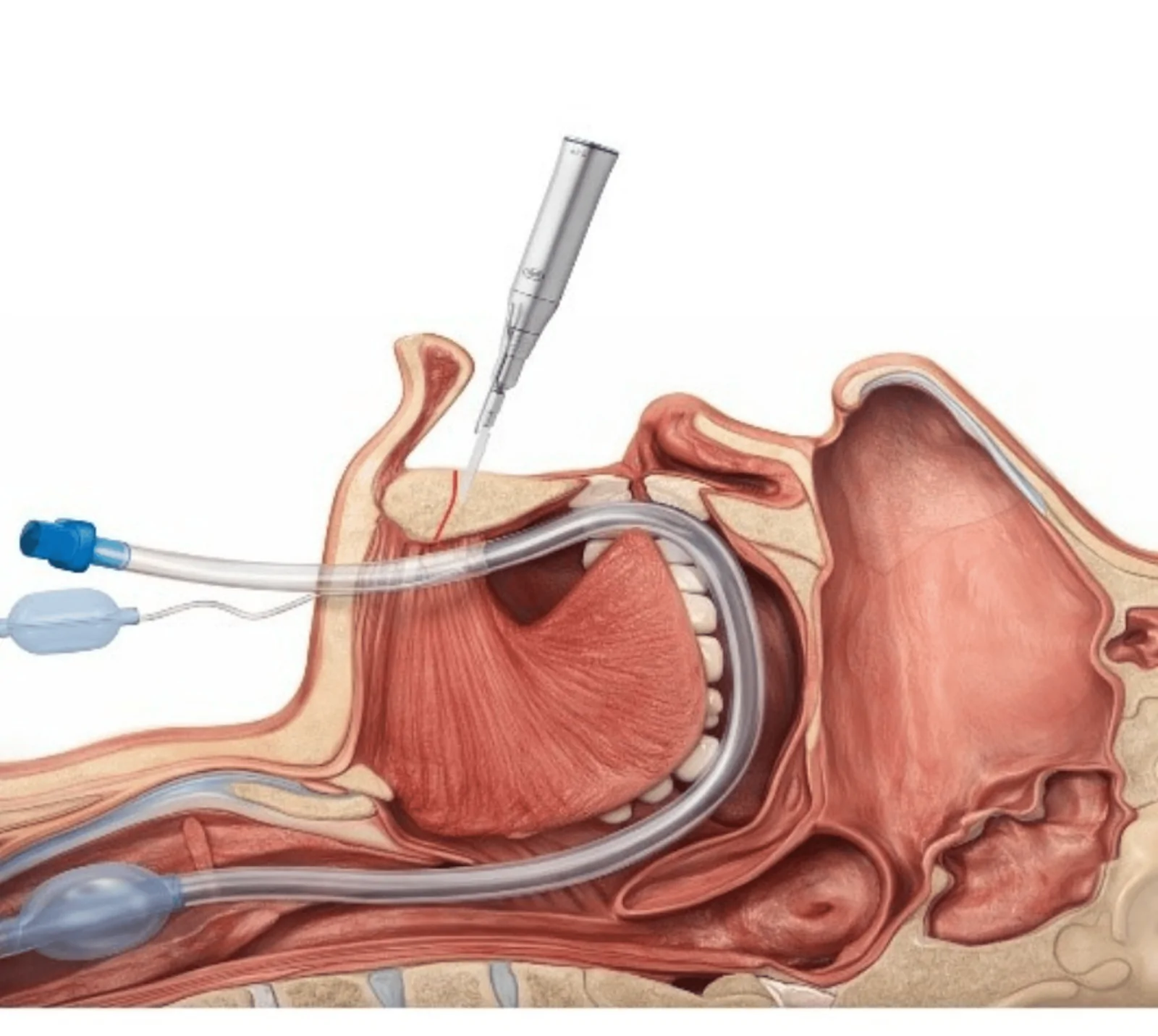
Illustration of the relationship between the endotracheal tube and the surgical field Created by the authors using Adobe Illustrator

**Figure 3:**
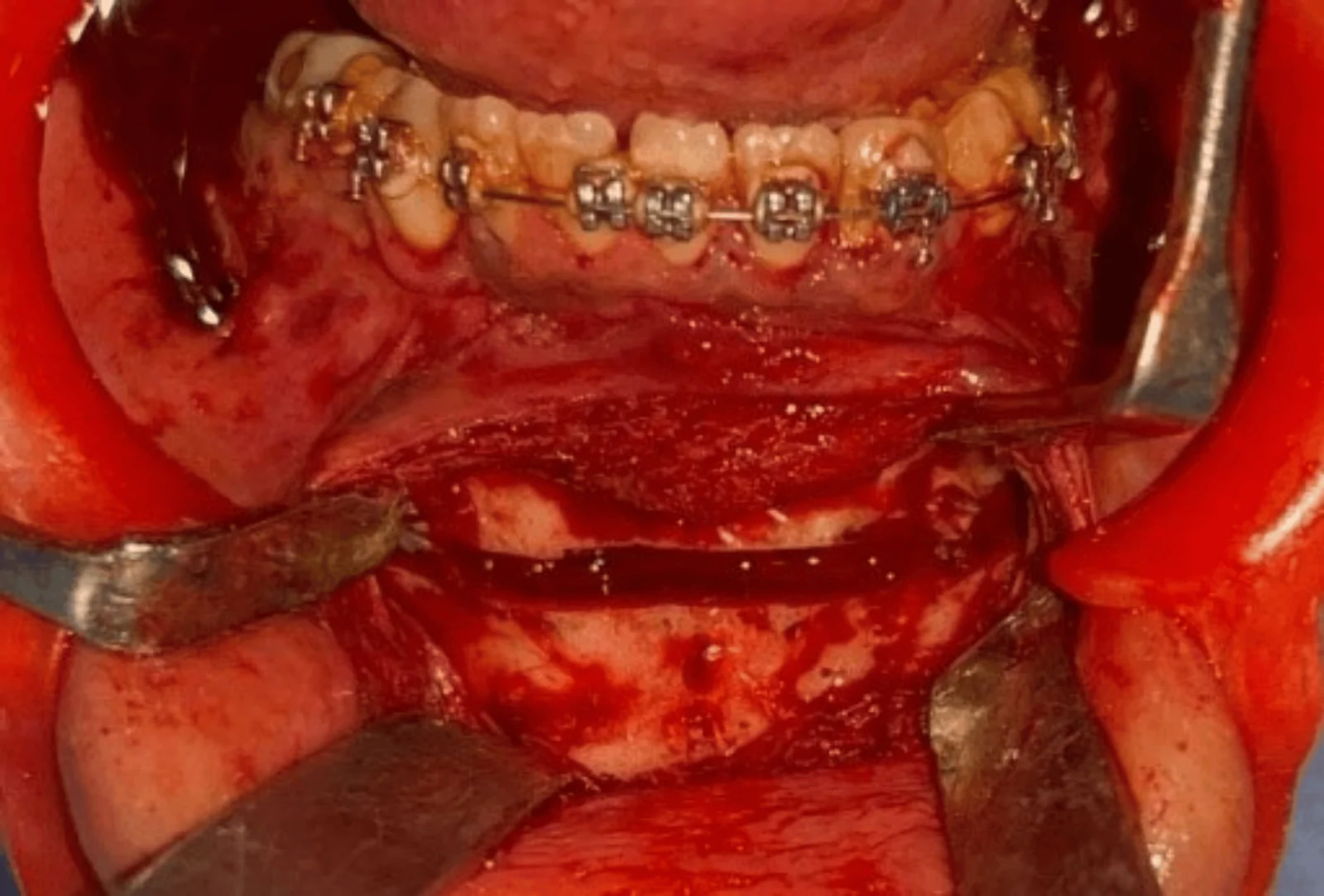
Genioplasty osteotomy site

The Le Fort I advancement and bilateral sagittal split osteotomy setback were completed uneventfully. During the genioplasty phase, while performing the horizontal osteotomy in the mandibular symphysis region using a powered oscillating saw (Figure 2), the anesthesiologist noted a sudden decrease in delivered tidal volume, an audible air leak, and a reduction in peak airway pressure. The capnography waveform became irregular, and the ventilator alarm indicated a circuit leak. Bubbling of air mixed with blood was observed at the genioplasty osteotomy site (Figure 3), raising suspicion of ETT damage.

Manual ventilation revealed increasing difficulty in maintaining adequate ventilation, with reduced chest rise and falling oxygen saturation.

Management

Surgery was immediately halted, and the surgical field was packed. The anesthesiology team switched to 100 percent oxygen and attempted to maintain ventilation manually. Direct inspection revealed partial transection of the ETT in the submental portion, likely caused by the osteotomy instrument.

**Figure 4:**
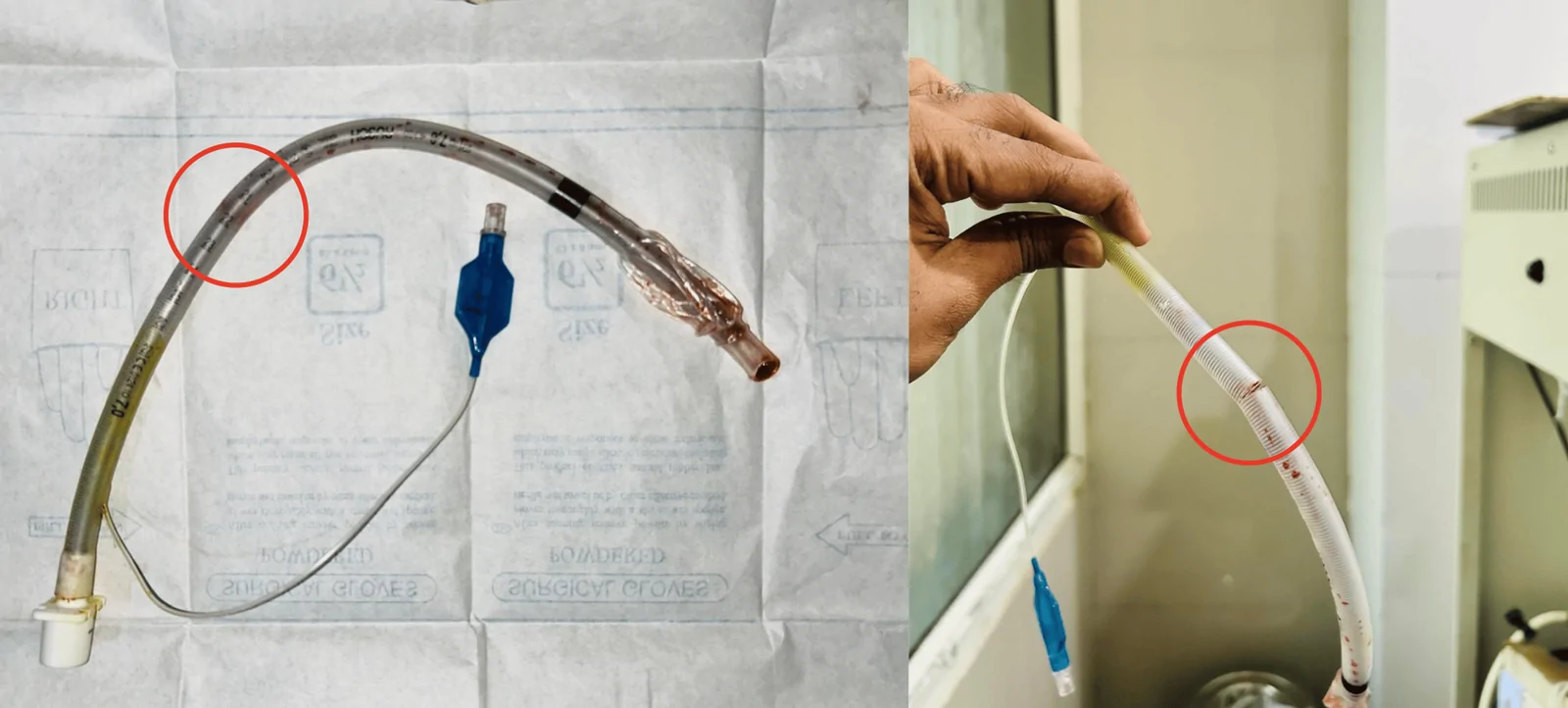
Partially transected endotracheal tube (marked within a red circle)

An emergency ETT exchange was performed under direct laryngoscopy with surgical assistance for tissue retraction. The damaged tube was carefully removed and replaced with a new cuffed ETT using a bougie as an airway guide (Figure 4).

**Figure 5:**
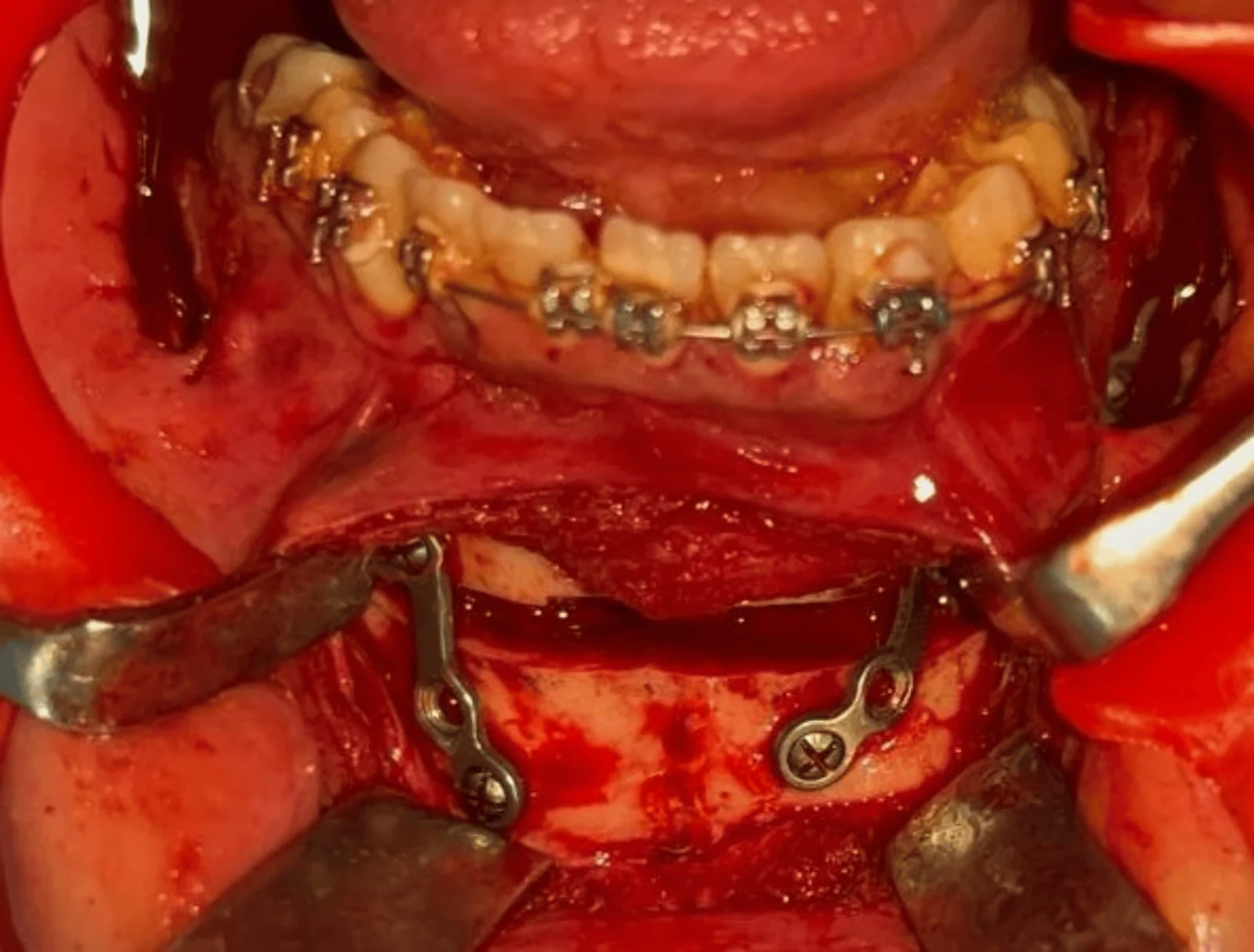
Fixation of the genioplasty segment

Tube placement was reconfirmed by capnography and auscultation. Oxygen saturation returned to normal, ventilation parameters stabilized, and surgery was resumed after ensuring hemostasis and airway security. The genioplasty was completed cautiously without further complications (Figure 5).

## Discussion

Accidental damage to the ETT is a rare but potentially life-threatening intraoperative complication most commonly encountered during head and neck and maxillofacial surgeries. These procedures involve a shared surgical-anesthetic airway and frequently require the use of powered instruments in close proximity to the airway, increasing the risk of inadvertent ETT injury [10,11].

Genioplasty poses a particular risk because the osteotomy is performed in the anterior mandible adjacent to the floor of the mouth and oral cavity, where the ETT may lie relatively unprotected. Limited working space, reduced visibility due to blood and irrigation fluids, and the use of oscillating saws further contribute to airway vulnerability [6].

Mechanisms of ETT injury reported in the literature include partial or complete transection by oscillating saws, osteotomes, rotary drills, and sharp retractors. Thermal injury from electrocautery and compression-related tube deformation have also been described [7,12]. Partial ETT damage is particularly hazardous because ventilation may initially appear adequate, delaying recognition until hypoventilation or hypoxia occurs [13].

Early recognition of ETT compromise is essential. Sudden reductions in tidal volume, audible air leak, altered capnography waveform, difficulty in ventilation, and unexpected oxygen desaturation are key warning signs. Continuous capnography is especially valuable, often indicating abnormalities before hypoxia develops [14]. Prompt communication between anesthetic and surgical teams is critical, and airway security must take precedence over surgical progress. Immediate suctioning and oral cavity packing reduce aspiration risk before airway intervention [15].

Management depends on the extent of tube damage and the patient's ventilatory status. Controlled tube exchange using direct or video laryngoscopy with a bougie or airway exchange catheter may be successful. However, anesthesiologists must be prepared for an emergency surgical airway if reintubation fails, particularly in the presence of bleeding and limited access [8,15].

Airway management for orthognathic surgery must reconcile secure ventilation with unobstructed surgical access. Nasotracheal intubation remains the standard approach, while oral intubation with submental conversion is a recognized alternative when a nasal tube would compromise the operative field or when concurrent nasal work is planned; tracheostomy is reserved for prolonged or complex reconstructions [3-5]. Instrument-related ETT injury is an uncommon but repeatedly reported complication of these procedures, with partial and complete transection following the use of oscillating and reciprocating saws, rotary drills, and Gigli saws during orthognathic and maxillary osteotomies [6-8]. Reinforced flexometallic tubes resist compression and kinking but, as in the present case, do not reliably withstand a direct strike from a powered saw. Associated intraoperative airway challenges include accidental extubation, tube kinking, cuff perforation, aspiration of blood and irrigation fluid, airway edema, and difficult reintubation, any of which may compound the primary injury and must be anticipated by the anesthetic team [7,12].

Several measures may reduce the risk of ETT injury during genioplasty and related osteotomies. The submentally routed tube segment should be shielded from the saw with a retractor or malleable protector, and its position confirmed immediately before each saw activation. Osteotomy depth should be controlled and the lingual cortex engaged under direct vision; where available, piezoelectric instrumentation, which cuts mineralized bone but spares soft tissue and the tube, offers an inherently safer alternative to conventional saws for cuts adjacent to the airway. Continuous capnography and close monitoring of ventilation parameters remain indispensable, as they frequently signal tube compromise before hypoxia develops [13,14], and the surgical and anesthetic teams should share an explicit, rehearsed plan for rapid tube exchange should injury occur [15].

This case illustrates how a routine orthognathic procedure can escalate within seconds into a critical anesthetic emergency, and how structured vigilance converts a potential catastrophe into a recoverable event. It also underscores that such injuries are largely preventable through simple, deliberate safeguards rather than reliance on chance.

## Conclusions

Intraoperative ETT injury during genioplasty, though rare, can progress rapidly to a life-threatening airway emergency. This case reinforces that early recognition through continuous capnography, disciplined communication between the surgical and anesthetic teams, and immediate readiness for tube exchange are decisive in averting harm. Equally important, the complication is largely preventable: shielding the submental tube segment, confirming its position before osteotomy, and considering piezoelectric instrumentation should be routine safeguards in shared-airway surgery. As a single case report, these observations are limited in generalizability, but they provide a practical framework for anticipating and mitigating a complication that is easily overlooked until it occurs.
